# Understanding the strategies employed to cope with increased numbers of AIDS-orphaned children in families in rural settings: a case of Mbeya Rural District, Tanzania

**DOI:** 10.1186/s40249-016-0233-7

**Published:** 2017-02-07

**Authors:** Nelsensius Klau Fauk, Silivano Edson Mwakinyali, Sukma Putra, Lillian Mwanri

**Affiliations:** 1 Institute of Resource Governance and Social Change, Jl. R. W. Monginsidi II, No. 2, Kel. Kelapa Lima, Kec. Kelapa Lima, Kupang, Nusa Tenggara Timur Indonesia; 2 National Food Reserve Agency, P.O. Box 5384, Dar es Salaam, Tanzania; 3 Binus University International, Jl. Hang Lekir I No. 6, Senayan, Jakarta, 10270 Indonesia; 4 Discipline of Public Health, School of Health Sciences, Flinders University, GPO Box 2100, Adelaide, 5001 South Australia Australia

**Keywords:** Adoptive families, AIDS-orphaned children, Coping strategies, HIV, AIDS, Mbeya Rural District, Tanzania

## Abstract

**Background:**

The purpose of this study was to understand the strategies employed by families that adopt Acquired Immune Deficiency Syndrome (AIDS)-orphaned children (Adoptive families) for coping with and mitigating the impact of AIDS in Mbeya Rural District, Tanzania. High numbers of AIDS-orphaned children aged below 18 years in Mbeya Region have led to increasing the burden of families caring for them. Understanding the coping strategies and impact mitigation activities employed by adoptive families is important in order to develop programmes to help them.

**Methods:**

This study employed a qualitative method for data collection (one-on-one in-depth interviews). The respondents included 12 male and 8 female heads of families that provide essential care for AIDS-orphaned children in Mbeya Rural District in Tanzania. The framework approach was used to analyse the data that were collected from 15 July to 15 August 2010.

**Results:**

The study findings revealed that adoptive families faced several challenges including financial constraints due to increased needs for basic essentials such as health care expenses, school fees and food. Further impacts on adoptive families included shortage of work opportunities and limited time to address these challenges. To mitigate these challenges, adoptive families employed a range of coping strategies including selling family assets and renting out parts of cultivable land for extra cash. Task reallocation which involved the AIDS-orphaned children entering the labour force was also employed as a strategy to mitigate challenges and involved de-enrolling of children from schools so they could take part in income-generating activities in order to earn supplementary family income. The creation of additional income-generating activities such as poultry farming were other coping mechanisms employed, and these received support from both non-governmental organisations (NGOs) and governmental organisations, including the Isangati Agricultural Development Organization (local NGO) and the local government respectively.

**Conclusions:**

The current study identified challenges that adoptive families as well as the AIDS-orphaned children themselves faced in Mbeya Rural District, Tanzania. Recognition of these issues highlights the need for targeted interventions to address the underlying social determinants of human immunodeficiency virus or HIV and AIDS in affected populations in order to prevent further imposition of social, cultural and economic disadvantages on families that provide care for AIDS-orphaned children and the children themselves. These findings may prove useful in provoking discussions that may lead to HIV/AIDS prevention and the development of broader mitigation strategies to alleviate the impact of this scourge on families and communities in rural Tanzania, and in similar settings across the world.

**Electronic supplementary material:**

The online version of this article (doi:10.1186/s40249-016-0233-7) contains supplementary material, which is available to authorized users.

## Multilingual abstracts

Please see Additional file 1 for translations of the abstract into the five official working languages of the United Nations.

## Background

Across the world, the human immunodeficiency virus and acquired immune deficiency syndrome (HIV/AIDS) have become some of the most formidable health scourges of our times. Among the adverse outcomes, HIV/AIDS have resulted in large numbers of orphaned children who need families and other institutions to provide the necessary care for them. The 2016 UNICEF report reveals that approximately 13.3 million children aged 0 to 17 years were without one or both parents due to AIDS by the end of 2014, and of these, 11.0 million lived in Sub-Saharan Africa (SSA) [1, 2].

In SSA, the number of AIDS-orphaned children has been reported to have increased considerably during the last two decades, from fewer than one million in 1990 to 3.0 million in 2000 and to 11.0 million in 2014 [1–4]. Compared to low-populated nations in Africa such as Swaziland, Namibia, Botswana and Lesotho, densely-populated countries including Kenya, South Africa, Uganda and Tanzania have very high numbers of AIDS-orphaned children, which pose a higher burden of care in these countries [1, 5].

Although the first HIV/AIDS case in Tanzania was reported in Kagera Region in 1983, the Njombe and Iringa regions (which are geographically far away from the initial case) had the highest prevalence rates of HIV/AIDS, accounting for 14.0 and 9.1% respectively in 2013 [6–8]. This reflects how quickly this infection can spread across communities and population groups within and between regions. In 2013, a higher prevalence of epidemic was observed among the populations living in urban areas than those living in remote areas with the rate of 7.2 and 6.2%, respectively [6, 9]. The 2013 data also indicated that women were more susceptible to the infection than men, with 6% of women compared to 4% of men infected [9]. However, overall, Tanzania has been successful in reducing the prevalence HIV/AIDS, from 8.0% in 1995, 6.5% in 2004, 5.8% in 2007 to 5.1% in 2012 [8–11]. As of December 2013, the estimated number of people living with HIV (PLWHIV) in the country was 1,411,829 [9].

Similar to trends around the world, behavioural, socio-cultural and biomedical determinants as well as income inequality and poverty have all been linked to the transmission of the epidemic in Tanzania. Partaking in unsafe sex, both vaginal and anal, with multiple sexual partners, has been identified as the main contributing factor for the spread of the infection in the country and in similar settings [9, 10, 12]. Economic pressures due to income inequality and poverty have also led people to engaging in risky sexual behaviours, as well as contributing to poor health outcomes among populations in the country, through poor food security and poor access to healthcare [9, 13–16]. Other studies have also identified other biomedical and environmental mechanisms through which transmissions can occur including unsafe blood transfusions, medical injections, low level of voluntary medical male circumcision, high prevalence of sexually transmitted infection, mother-to-child transmission of HIV, high levels of couple discordance and patients’ low level of knowledge of HIV status [6, 8].

One of the impacts of the HIV/AIDS epidemic on the population in Tanzania is an increase in parental mortality rate which has led to increased numbers of AIDS-orphaned children [9]. A report by Leach [17] stated that AIDS-orphaned children account for approximately half of all orphans in Tanzania. Since HIV/AIDS-related deaths in parents continue to occur, it is estimated that the number of AIDS-orphaned children in the country will only continue to increase in the coming years. For example, it has been reported that the number of AIDS-orphaned children increased from less than a million in 2010 to 1.3 million by the end of 2013 [1, 18], and HIV-positive children are highly prevalent in the country. Of the total number of PLWHIV in Tanzania, 28% are children aged 0 to 14 years and 11.2% are young people aged 15–24 years [9].

Mbeya Region had the highest prevalence of HIV/AIDS in Tanzania accounting for 13.5%, which was significantly higher than the national rate of 7% (2007 figures) [7]. Although the region has succeeded in reducing the prevalence of HIV/AIDS to 9.0% by the end of 2012 [6], it still suffers the loss of the prime age group who are needed in the workforce and consequently for economic development [12]. Mbeya Region continues to have the highest numbers of AIDS-orphaned children aged below18 years in the nation. Without parental care or appropriate care, orphaned and vulnerable children (OVCs) are likely to face poor health and malnutrition, as well as socio-economic and cultural problems including inadequate schooling, migration, homelessness, social exclusion, abuse and exploitation [19, 20].

A number of initiatives have attempted to address the issues encountered by AIDS-orphaned children including those initiated by the Tanzanian government in collaboration with nongovernmental organisations (NGOs) at the national and local levels, including in Mbeya [9]. These have included addressing aspects of healthcare provision, such as general access to voluntary counselling and HIV testing, education and vocational training [21, 22]. For example, reports by the Tanzania Commission for AIDS indicate that each district of the Mbeya Region has NGOs supporting OVCs through material aid such as school uniforms, books, school fees, vocational training, and medical treatment [7, 9]. The scope of this assistance, however, is limited as there is a relatively small number of NGOs and the majority of adoptive families still face many problems including having to pay for the sudden increase in essential family needs such as food, clothes and school fees.

Despite the serious impact of HIV/AIDS on adoptive families, limited studies have reported on the effective strategies adopted by such families to mitigate the impact of AIDS. Understanding the coping strategies and impact mitigation activities employed by families that adopt AIDS-orphaned children to address emerging challenges is necessary in order to provide evidence that can inform programmes to help such families.

The aim of this study was to understand the coping strategies and impact mitigation activities employed by families that adopt AIDS-orphaned children in the Mbeya Rural District of Mbeya Region in Tanzania.

## Methods

### Study setting

This study was conducted in Mbeya Rural District in Mbeya Region which is located in the southern part of Tanzania. With a population of 305,319 and an annual growth of 3.1% as reported in the 2012 census [23], the Mbeya Rural District covers an area of 19,093 km^2^, comprising 572,089 ha of arable land. Of this, 211,420 ha are used for agriculture, 46,623 are reserved for forestlands, 500 are game reserve areas and the rest are water areas and used for residential dwellings [24]. The majority of people in Mbeya Rural District (84%) are small-scale farmers, with a small proportion of their income being generated by animal husbandry, hunting and small businesses [23, 24]. Charcoal processing is also a practised income generating activity among Mbeya Rural District inhabitants. The district has 158 villages comprising 74,268 households with an average of four people per household [23, 24].

### Theoretical frameworks

The current research was guided by several theoretical frameworks including the social network theory (SNT) [25, 26], which examines the characteristic patterns of ties between actors in a social system rather than the characteristics of the individual actors themselves. Using the SNT, we analysed Mbeya Rural District community and its composition, assuming that the social structure of this community was itself largely responsible for determining individuals’ behaviours and attitudes, for example to care for orphaned children whose parents were part (actors) of these communities.

It is well acknowledged that African culture (including Tanzanian culture) is often communitarian, where the idea of community is central [27–30]. The African communal way of life and social networking, brings together family members, relatives and other people who consider themselves to be part of ‘a community’ to become involved in communal events which may include – as is the case in the current study – the provision of care to HIV/AIDS-orphaned children.

It has been documented that African communities celebrate, mourn and perform essential duties and rituals together, and value human relationships over material possessions with a sense of pride [27, 31]. Several authors in African studies have suggested that this sense of community and human relationships has its roots in the African moral ethos of ‘*Ubuntu’ or ‘Afro-communitarianism’ – the idea that*_*‘*_ *a person is a person through other people’* [15, 27, 29–32]. Afro-communitarian values are based on a consideration that an individual is an integral part of the family and the community, and that every individual has the moral obligation to be concerned for the good of others in the same communal framework [30].

The influence of Afro-communitarian ethos is evident in the way many African communities, including those in the Mbeya Rural District, care for orphaned-children, the numbers of which have increased following the HIV/AIDS epidemics which have hit many communities. Afro-communitarian theory suggests that in the event that a member of a family is deceased (including from HIV/AIDS), close and extended family members consider it their responsibility to take care of the orphans left behind. Anecdotal evidence indicates that other distant community members would often offer many forms of help to the bereaving families and children making sure that the burden of loss is mitigated. The Afro-communitarian sense of community also influences how care is provided to orphans, which includes the adoption of orphaned children by family members. Likewise, there are suggestions that life in African communities is based on the philosophy of live-and-let-live [28]. People may help one another without expecting remuneration. This Afro-communitarian way of good human relations is the most important and valuable attitude and should be encouraged, particularly in relation to providing effective care in a society affected by HIV/AIDS, where resources are scarce.

These social fabrics and characteristics of African communities are similar to what is known in new public health theories as ‘community social capital’, which has been identified as a strong protector of health [14, 33], especially where community social capital values provide supportive environment to positive health outcomes. Similar to available evidence from the literature where social capital has been stated to contribute to population wellbeing, it is plausible to state that in settings such as the Mbeya Rural District where it is practised, community social capital contributes to the wellbeing of AIDS-orphaned children [33].

### Study method and data collection

Given the limited understanding of the relationship between the impact of HIV/AIDS and local social, cultural and economic factors in heavily affected poor rural settings, a qualitative inquiry was employed in this study. Qualitative methods are able to generate information on how social environments influence health-related behaviours and strategies [34].

Data collection involved conducting one-on-one in-depth interviews with heads of families that had adopted AIDS-orphaned children. Purposive sampling was employed to recruit participants who met the following inclusion criteria:(i) caring for five or more AIDS-orphaned children; (ii) aged 55 years or above; and (iii) being very poor and owning meagre household assets. Participants bearing these criteria were bearers of the highest burden of caring for AIDS-orphaned children in these settings, and were considered to be the most vulnerable groups impacted by the AIDS scourge. Understanding their situations and needs is important in order to develop programmes that can support them. Thirty-three potential participants met the inclusion criteria and were sent an invitation letter through Isangati Agricultural Development Organization, a non-governmental organisation providing poultry project to help families that adopt AIDS-orphaned children to cope with and mitigate the impact of AIDS. Of the 33 invited, only 20 heads of families comprising 12 female and 8 male responded and participated in the study. An interview with each participant was scheduled at a convenient time for them and was conducted in their houses. The interviews were conducted in Swahili and recorded using a tape recorder after consent was given by each participant. Each interview took between 45 and 90 min. This study was conducted from 15 July to 15 August 2010.

### Data analysis

Interview data were transcribed verbatim and translated from Swahili to English by one of the authors of this paper (SEM) and checked for accuracy by another author (LM); both authors are fluent in both English and Swahili. English transcripts were imported into the qualitative data analysis software package NVivo 9 (QSR International Pty Ltd. Doncaster, Victoria, Australia). The framework approach described by Ritchie and Spencer [35] was used to analyse the data. It involves a systematic approach to data management in order to provide coherence and structure to the qualitative data [35, 36]. Passages of text representing repeated themes were identified and assigned headings according to the context, and then coded to as many relevant categories as possible to reduce the likelihood of missing key points. The data were then synthesised in a chart using headings identified in the thematic analysis [35]. This approach enhances rigour, transparency and validity of the analytic process [37]. Analysis was both deductive, with categories derived using prior knowledge, and inductive, with categories emerging purely from the data [38].

## Results

From the analysis of interview transcripts, the following adoptive families’ coping strategies and mitigation activities were identified: (i) selling of family assets, (ii) renting out land, (iii) reducing the size of the family’s cultivable land, (iv) reallocation of labour, and (v) withdrawing children from school. Assets, land ownership and gender inequalities played an important role in these settings and were categorised as a standalone theme. The details of the six identified themes are described further below.

### Selling of family assets

Most participants reported the inability to provide and sustain families due to additional expenditure imposed by adopting AIDS-orphaned children. In order to address the supplementary family needs including food and medical costs, adoptive families sold some of their assets such as radios, bicycles and livestock.

*“When it comes to the needs of the children in the family, I would do anything. I have sold a few assets including livestock, oxen and radio. The family needs things like food and health care, which is something urgent, important and cannot be postponed. I either try to provide a solution or be ready to accept possibly the worse outcome.”* (A 60-year-old male taking care of seven orphans)

*“To cope with our* [the family head’s and the children’s] *daily needs, I had to sell a few things left by my husband before he died, including bicycles and livestock. Food is a necessity and it has to be there when the kids are hungry. It is different to the needs for new clothes or school fees. I can tell any of my children that I do not have money to buy her or him new clothes, but I cannot tell them this when it comes to food. I have experienced even worse situations when one of my children* [orphans] *got sick. I had to think of the need for food and health care while I did not have much to cover either …….”* (A 61-year-old female taking care of five orphans)

### Renting out land

The majority of families were small-scale farmers and their agricultural land was the major source of family livelihood. These findings showed that renting out land was one of the strategies employed to cope with the families’ increasing needs.

*“I used to cultivate two pieces of land but for the last few years, I have been renting out the bigger one in order to get some cash … but I still cultivate the smaller one.”* (A 63-year-old male taking care of eleven orphans)

“*In rural areas without land you are nothing because we mainly depend on farming activities and land is the only asset we have been given by God.”* (A 56-year-old female taking care of eight orphans)

### Reducing the size of the family’s cultivable land

Our findings also indicated that female-headed families were more disadvantages and had to further reduce the size of their cultivable land in order to address the emerging shortage of workforce, and to be able to spend more time providing essential lifesaving care to AIDS-orphaned children. All female participants indicated that they had to reduce their cultivable land in order to spend less money on farm necessities and on hiring labour. They also expressed spending less time on agricultural activities than they would normally do due to the overwhelming responsibilities related to the provision of care to AIDS-orphaned children.

*“After the death of my son and his wife, I took their five children. My son was helping me to buy things for the farm and to hire labour. But now I decided to reduce the farm size from one acre to half an acre because I don’t have anyone to help me. Now I don’t have much time to spend on the farm as I normally used to; most of the time I am at home looking after my grandchildren. This affects my crop yields as I am not harvesting like before. Reduction of cultivable land is the only option for me to cope with labour shortage and time; I cannot leave my grandchildren alone because they need a lot of attention and I don’t have money for hiring labour for my farm.”* (A 62-year-old female)

*“… I do not reduce it* [the size of the cultivable land] *because it will also reduce the crops I produce…”* (A 60-year-old male taking care of seven orphans)

Although the majority of female participants seemed to be aware of the negative impacts of reducing cultivable land through selling or renting, they did not have alternative choices to address their circumstances. The reduction of cultivable land subsequently had food and livelihood implications, and would usually lead to hunger and food insecurity.

*“I have experienced that reducing cultivable land by an acre definitely reduces the crops I produce at harvest time. They will not last long. It is just not enough for children and me. And most of the time I have to think of other ways to get money to buy food…”* (A 59-year-old female taking care of six orphans)

The study elucidated that some families, especially those headed by females, opted to work elsewhere than in farming in order to generate more soft income for family needs. However, because the alternative jobs including casual work and seasonal petty businesses were low paying, these families continued to struggle to make the ends meet.

*“I have been involved in casual work way too often. Even though I get paid less, it helps us* [the family head and her children] *survive. The cash I get is just used to buy food and cover other needs”* (A 66-year-old female taking care of seven orphans)

### Reallocation of labour

Due to a high dependency ratio involving a large family size, comprising children and the elderly, there was a reduction in the number of people in the working age group who provide the prime labour pool for families. In addition to low human resources for labour, families experienced high socio-economic and cultural burdens due to raising AIDS-orphaned children, which took the necessary energy and time needed to generate income. The poor labour force has implications and negative effects on the ways families implement their coping strategies. As a way of mitigating these effects, children were enlisted to participate in income-generating activities, including in raising poultry in the house. Two participants exemplified these assertions in the following statements.

*“It is rather good to have a small project in the family, which demands less labour and time so that I can take advantage of the children I have in order to solve the labour shortage.”* (A 60-year-old female taking care of five orphans)

*“… I find it very difficult to do everything alone. So I often ask two of my children to help me out. My son sells vegetables and my daughter takes care of the poultry at home.”* (A 67-year-old male taking care of six orphans)

Readjusting including minimising the time to work on farms, hiring labour from neighbours, using child labour, moving farming work closer to the family dwelling and cultivating short- term and drought resistant crops, such as cassava and sweet potatoes which can be ploughed multiple times in a year, were some of strategies adopted by adoptive families to address low human resource and its consequences.

*“I do not have much time and energy to cultivate labour-intensive crops like I used to do … I still do but it is a small one* [size of the area under cultivation]. *Now my children and I grow vegetables just next to our house. It is a bit easier for me to do and the kids can also take care of them.”* (A 64-year-old female taking care of eleven orphans)

*“Since last year, I started to grow more cassava and potatoes because they are drought resistant and I don’t have to take care of them every day. It seems good because I can adjust the time needed for working and for taking care of my children* [orphans].*”* (A 60-year-old male taking care of seven orphans)

### Withdrawal of children from school

Because of serious AIDS-related consequences in adoptive families, especially those headed by females, children were de-enrolled from school to provide needed workforce to produce for living and for necessary school expenses. In contrast to male-headed adoptive families, all female-headed adoptive families reported to have- temporarily or sometimes permanently-withdrawn children from school due to financial constraints and to cover for labour shortages. Withdrawing children from school was also an important strategy to reduce the school requirements and to allow savings from these to cover other essentials. Children took adults’ roles including keeping and selling poultry, selling vegetables and snacks in the streets, and engaging in casual work.

*“It is difficult to handle everything just by myself. I withdrew my older boy from school to help me out with taking care of the younger children in the family while I am working. He also helps me with taking care of poultry; sometimes he sells vegetables and gets some money.”* (A 62-year old female taking care of twelve orphans)

*“I have eight grandchildren who are in primary school, and now is a new school term and I am supposed to pay school fees and buy exercise books and pens for all of them. Two of them don’t have school uniforms. When I looked at my saving, I realised I don’t have enough money for sending all eight children to school and buying uniforms. If I will send all of them to school, then I will not have enough money to feed them. So I have decided to send two children who are in their final year and I will send the others after getting enough money. Because this is harvesting period, my children and I expect to get casual work on farms to harvest crops. From this work, I will be able to raise some money to send my grandchildren to school. But also in some farms, they don’t allow children and old people like me to work because we are considered slower than young labourers or those who are in productive age. That is why some farms owners do not want to spend their money on hiring labourers like us.”* (A 67-year-old male taking care of eight orphans)

Of particular interest is the finding that AIDS-orphaned female children in adoptive families engaged in high-risk work to earn the hard needed money for their adoptive families. In the female-headed adoptive families, engaging in sexual activity was often viewed as an opportunity to make quick money. Some female participants acknowledged the fact that their female AIDS-orphaned girls engaged in sex for money so that they could buy their own stuff and other family needs. Due to the lack of protection, AIDS-orphaned girl children were thus at a higher risk of unwanted pregnancies and STIs including HIV.

*“I am staying with seven orphans, four girls and three boys, who are all my grandchildren. Three months ago, I realised that three of my granddaughters (one who is 16 years old and the two who are 15 years old) got pregnant and did not know who were responsible for their pregnancies. It is difficult for me to blame them because I do not have money to give them to buy clothes, body lotion and other things. They used to go to the weekly market which is held once a week on Saturday and came back on Sunday with money, sugar, salt and other items, which they did not show me. The market attracts business people from the town and nearby villages. It is difficult for me to know with whom they got involved. One day I asked one child who was responsible for her pregnancy, and she told me that she did not know and threatened to poison herself and follow her parents if I kept on asking. I am expecting that in the next four months, one of the three children will give birth.”* (A 61-year-old female taking care of seven orphans)

### Assets, land ownership and gender inequality

Similar to many settings in developing countries, the communities in Mbeya Rural District are male dominated. As such, women face more challenges and have ‘less right’ to make family decisions, with subsequent implications on AIDS-orphaned child caring. Compared to female-headed adoptive families, the decision to sell family assets is easier in male-headed adoptive families. Although a few female heads of adoptive families reported to have sold family assets, they needed permission from their dead husbands’ male relatives.

*“My husband left me bicycles, livestock and also land, but to use or sell them I have to get permission from his relatives. Here everything* [assets] *in the family belongs to men* [the husbands]*… After he* [her husband] *passed away, his relatives always want to know about all these: the land, livestock, and so on.”* (A 61-year-old female taking care of five orphans)

*“… I have a plough. If it is allowed, then I want to sell it because I do not use it that often … I first have to get permission from my husband’s male relatives before selling the asset.”* (A 64-year-old female taking care of eleven orphans)

Inequalities in assets and land ownership were additional factors contributing to the challenges faced by adoptive families, particularly those headed by females. The majority of the female participants stated they had no power and control regarding their cultivable land and some of the family assets. The husband’s relatives controlled the land even though these relatives did not contribute to the day-to-day caring of the AIDS-orphaned children. In every season female heads of adoptive families needed to request permission to use the land for the family’s livelihood. On the contrary, the male heads of adoptive families owned large pieces of land and had control over what they could use the land for.

*“Family land mostly belongs to the men, so even to cultivate the land of my late husband I have to ask for permission from my husband’s relatives and it should be done under their control. I cannot rent out the land either unless it is done under the control of his relatives because people have to deal with the land owners. I am not considered a land owner of my husband’s land even though he has died”* (A 58-year-oldfemale taking care of nine orphans)

“*Although we have been given the gift of land by God, our society doesn’t give us the right to own land … I have rented out the land in the last few seasons because the family was in need, but I did it under the control of my husband’s relatives.*” (A 56-year-old female taking care of eight orphans)

## Discussion

By examining the experiences of care provision to AIDS-orphaned children, the current study provides information that can be used to improve our understanding about coping strategies employed by adoptive families to address the impact of AIDS and AIDS-orphaned children on their households.

Consistent with previous literature [39–41], the current study suggests that to mediate these impacts, families adapt various coping strategies including the selling of family assets and implementing small-scale projects such as poultry farming. Findings from previous studies [41–44] have indicated that the sale of family assets including land, bicycles and radios, leads to unsustainable achievements, as most families end up becoming asset poor, which often exacerbates the impact of AIDS on these families. Renting out land as a strategy for coping with the impact of AIDS as reported in this study has also been described in previous studies [45–47]. However, in the socio-cultural context of African communities, in which men dominate and are recognised as owners of family assets, female-headed adoptive families often tend to be disadvantaged. For example, females, unlike males, have to ask for permissions to sell/rent assets and land from their deceased spouse’s male relatives [47].

The reduction of land used for cultivation and reallocation of labour have been reported [43, 48, 49] as additional mitigating and coping strategies used by adoptive families to address time constraints and low human resource for labour issues. Similar practices were observed in the current study. These strategies were used to minimise the time spent working on farms in order to create time needed to provide essential care to AIDS-orphaned children, who at times needed intensive caring, especially when they were unwell. Our research findings are also consistent with evidence suggesting that addressing the impact of AIDS on adoptive families leads to further consequences including a decrease in crop yields and a decline in the range of crops grown for food and revenue [42, 44, 50].

The reduction in education opportunities for AIDS-orphaned children and the emergence of child labour - while problematic - were seen as necessary strategies for coping with the impact of AIDS. For example, older children were de-enrolled from school so that they could care for the others, usually younger children or work on the farm to supplement income to meet adoptive families extended needs. Similar findings have also been noted elsewhere [51–56], suggesting unequal distribution of disadvantages mainly skewed to poor families, especially those located in poor rural settings. The detrimental effects resulting from withdrawing children from school and the use of AIDS-orphaned child labour have been documented in several studies and have led to further problems such as poor child development [57–60].

Similar to findings reported elsewhere [42, 61, 62], the current study confirms that children, especially girls, engage in transactional sex in order to generate the money needed for basic essentials in their new adoptive families. These practices have been noted to increase female children’s vulnerabilities including unplanned pregnancies and acquisition of STIs including HIV. These factors lead to a vicious cycle of disadvantages in affected families, communities and nations.

## Conclusions

The current study provides information regarding strategies employed to address and mitigate the impact of AIDS on adoptive families in Mbeya Rural District, Tanzania. The selling of family assets, renting out of family land, reducing the size of the family’s cultivable land, reallocation of labour and withdrawal of children from school were some of the strategies employed to create additional resources needed to meet for the families’ extended essential basic needs.

Despite the existence of strong ‘African community social capital’ provided by socio-cultural norms of the African culture, where ‘*Ubuntu’* or ‘Afro-communitarianism’ provides protection for *‘the needy’* in the community, issues of land ownership and gender inequality seem to be problematic in Mbeya Rural District [47]. Our findings demonstrated an unequal distribution of power for decision-making between female-headed and male-headed households, which influenced the ways families coped and mitigated the impact of AIDS with female-headed households being unequally disadvantaged.

While the findings of the current study provide significant insights regarding socioeconomic and cultural strategies adopted by adoptive families to cope with the impact of AIDS-Orphaned children, the study has a few strengths and limitations. The use of a qualitative approach in this study provided an in-depth and highly contextualized information and insights into pertinent issues including those affecting AIDS-adoptive families and AIDS-orphaned children. It also involved adoptive families that varied in leadership including female-headed and male-headed families, providing further in-depth understanding of socio-cultural and gender dynamics and the impact of these in coping with the AIDS related issues in the study setting. Recognition of these issues highlights the need for targeted interventions to address the underlying social determinants of health in these populations and to further prevent the imposition of social, cultural and economic disadvantages on adoptive families. The current findings are also useful for provoking discussions that may lead, not only to HIV/AIDS prevention, but also to mitigating the impacts of these and similar emerging health and social issues in affected families and communities. The study limitations include the inability to offer generalisation of these issues to the whole of Tanzania outright. However, we believe that lessons learned from these findings can be transposed to similar settings in Tanzania and beyond. Further studies are recommended to elucidate issues that the current study could not provide.

## Additional file

Additional file 1: Translation of the abstract into the five official working languages of the United Nations. (PDF 423 kb)

## Abbreviations

AIDS: Acquired immune deficiency syndrome
HIV: Human immunodeficiency virus
NGO: Non-governmental organisation
OVCs: Orphaned and vulnerable children
PLWHIV: People living with HIV
SNT: Social network theory
SSA: Sub-Saharan Africa
STI: Sexually transmitted infection
