# Supplementary material for: Understanding the strategies employed to cope with increased numbers of AIDS-orphaned children in families in rural settings: a case of Mbeya Rural District, Tanzania
Source: Infect Dis Poverty. 2017 Feb 7;6:21. doi: 10.1186/s40249-016-0233-7 (PMC5297121; doi:10.1186/s40249-016-0233-7)
Supplement: Additional file 1: — Translation of the abstract into the five official working languages of the United Nations. (PDF 423 kb) [file 40249_2016_233_MOESM1_ESM.pdf]

إستراتيجيات الأسر المتبنية لمواجهة وتخفيف آثر الإيدز على الأطفال الأيتام المصابين بمرض الإيدز في منطقة مبيا بدولة تنزانيا

نلسينسياس كلو فوك، سيليفانو إدسون مواكينيالي، ليليان موانري

الملخص

الأهداف: **استهدفت الدراسة فهم الإستراتيجيات المستخدمة من قبل الأسر المتبنية للأطفال الأيتام المصابين بمرض الإيدز (نقص المناعة المكتسبة) لمواجهة وتخفيف آثر الإيدز على هذه الأسر في منطقة مبيا الريفية بدولة تنزانيا.**

الأساليب: **استخدمت هذه الدراسة تحقيق نوعي لجمع البيانات باستخدام المقابلات المتعمقة المباشرة. واشتملت الدراسة على مشاركين من أرباب الأسر من الذكور والإناث الذين قدموا الرعاية الأساسية للأطفال الأيتام المصابين بمرض الإيدز في منطقة مبيا الريفية بدولة تنزانيا. واستخدم تحليل البيانات النهج الإطاري.**

النتائج: **واجهت الأسر المتبنية لأطفال أيتام مصابين بمرض الإيدز العديد من التحديات بما في ذلك القيود المالية بسبب زيادة نفقات الرعاية الصحية والطلب على الغذاء داخل الأسر. واشتملت الآثار الإضافية لمرض الإيدز بين الأسر المتبنية لأيتام مصابين بمرض الإيدز على نقص فرص العمل وضيق الوقت للتصدي للتحديات الناشئة. وللحد من هذه التحديات، استخدمت الأسر المتبنية لأيتام مصابين بمرض الإيدز مجموعة من الإستراتيجيات للتعامل مع هذه المواقف بما في ذلك بيع أصول الأسرة وتأجير قطع من الأراضي الصالحة للزراعة لجني مزيد من الأموال. كما تم استخدام أيضًا إستراتيجية إعادة توزيع المهام باستخدام القوى العاملة من الأطفال الأيتام المصابين بمرض الإيدز للتخفيف من حدة التحديات، كما يتم إلغاء التحاق الأطفال بالمدارس للمشاركة في الأنشطة المدرة للدخل لدعم دخل الأسرة الإضافية واحتياجاتها. وقد حصلت آليات التكيف الإضافية لخلق مزيد من الأنشطة المدرة للدخل، مثل مشاريع الدواجن، على الدعم من كل من المنظمات غير الحكومية والمؤسسات الحكومية بما في ذلك منظمة التنمية الزراعية إسانجاتي (IADO) والحكومة المحلية.**

الاستنتاجات: **تقدم هذه النتائج معلومات حول التحديات التي تواجهها الأسر المتبنية لأيتام مصابين بمرض الإيدز وكذلك الأطفال الأيتام المصابين بمرض الإيدز أنفسهم في منطقة مبيا الريفية. وإدراكًا لخطورة هذه المشكلات، هناك دعوات لضرورة إجراء تدخلات هادفة لمواجهة المحددات الاجتماعية الكامنة وراء فيروس نقص المناعة البشرية أو HIV والإيدز في السكان المتضررين لمنع مزيد من التدهور في الحرمان الاجتماعي والثقافي والاقتصادي المفروض على الأسر التي تقدم الرعاية للأطفال الأيتام المصابين الإيدز والأطفال المتضررين أنفسهم. قد تكون هذه النتائج مفيدة في إثارة مناقشات قد تؤدي إلى الوقاية من فيروس نقص المناعة البشرية HIV / الإيدز، ووضع إستراتيجيات تخفيف على نطاق أوسع للتخفيف من آثر المرض على الأسر والمجتمعات الريفية في تنزانيا والبيئات المماثلة في مختلف أنحاء العالم.**

Translation of the abstract into the five official working languages of the United Nations

坦桑尼亚姆贝亚农村地区收养 AIDS 孤儿家庭对 AIDS 孤儿的应对和减轻 AIDS 影响的策略

Nelsensius Klau Fauk, Silivano Edson Mwakinyali, Lillian Mwanri,

摘要

引言：本研究旨在了解坦桑尼亚姆贝亚农村地区为应对和减轻收养AIDS孤儿的家庭因AIDS对家庭的影响所采取的相关策略。
方法：本研究采用一对一深度访谈收集数据的方式进行定性研究。受访者包括坦桑尼亚姆贝亚郊区为 AIDS 孤儿提供基本护理的男性和女性家长。以框架做数据分析。
结果：AIDS 孤儿收养家庭面临众多挑战，包括由于不断增长的健康保健支出和食品消费需求。艾滋病对收养家庭的进一步影响包括缺乏就业机会，并需在有限的时间应对紧急情况。AIDS 孤儿收养家庭采用多种策略来应对上述状况，包括出售家产、出租部分耕地以换取额外的资金。AIDS 孤儿作为劳动力进行工作重新分配也是策略的一部分以减轻以上状况，包括学龄儿童辍学参与有偿活动以贴补家用。而创造额外的有偿活动，如家禽养殖是其他应对机制，获得了非政府组织和政府部门，包括 Isangati 农业发展组织和当地政府的大力支持。
结论：本研究确定了坦桑尼亚姆贝亚郊区AIDS孤儿收养家庭和孤儿自身所面临的众多挑战。认识这些社会问题，采用必要的干预措施以应对受HIV/AIDS影响的人群的潜在社会决定因素，从而预防收养家庭和孤儿自身受到社会、文化和经济方面更深的伤害。这些问题值得深入研讨，有助于HIV/AIDS的预防，有助于制定更好的策略来减轻坦桑尼亚及全球其他情况艾滋病对家庭和社区的影响。

Translated from English version into Chinese by Jian-Hai Yin, edited by Pin Yang

CDC CHINESE CENTER FOR DISEASE CONTROL AND PREVENTION
NATIONAL INSTITUTE OF PARASITIC DISEASES

Les stratégies mises en place par les familles adoptives d'orphelins du SIDA pour gérer la maladie et minimiser l'impact de celle-ci, dans la région de Mbeya, en Tanzanie

Nelsensius Klau Fauk, Silivano Edson Mwakinyali, Lillian Mwanri,

Résumé

Objectifs : cette étude, réalisée dans la région de Mbeya, en Tanzanie, avait pour but de comprendre comment les familles adoptives d'orphelins du SIDA (syndrome d'immunodéficience acquise) gèrent la maladie et minimisent l'impact de celle-ci sur leur famille.
Méthodes : une enquête quantitative a été menée dans le cadre de cette étude afin de collecter des informations grâce à des entretiens individuels. Les personnes interrogées sont des pères et mères de famille de la région de Mbeya, en Tanzanie, ayant pris en charge des orphelins du SIDA. L'approche cadre a été utilisée pour l'analyse des données.
Résultats : les familles adoptives d'orphelins du SIDA font face à des nombreuses difficultés, notamment des difficultés financières dues à des frais médicaux grandissants et aux besoins de la famille en nourriture. Mais le VIH impose d'autres défis à ces familles adoptives d'orphelins du

SIDA : elles ont plus de mal à trouver du travail et peu de temps à consacrer aux problèmes qu'elles doivent affronter. Afin de surmonter ces difficultés, les familles adoptives d'orphelins du SIDA ont trouvé des solutions, lesquelles incluent la vente de biens appartenant à la famille ou la location de parcelles de terrer cultivables. La redistribution des tâches par le travail des orphelins du SIDA est également une stratégie utilisée par ces familles. Les enfants sont déscolarisés afin de participer à des activités génératrices de revenus, leur permettant ainsi de soutenir la famille et ses besoins sur le plan financier La création d'activités supplémentaires créatrices de revenus, telles que les projets d'élevage avicole, est devenue un mécanisme d'adaptation aujourd'hui soutenu par des organisations gouvernementales et non gouvernementales telles que l'Organisation pour le développement de l'agriculture d'Isangati (IADO) et les autorités locales.

Conclusions : ces résultats sont révélateurs des défis auxquels doivent faire face les familles adoptives d'orphelins du SIDA mais également les orphelins eux-mêmes dans la région rurale de Mbeya. La découverte de ces éléments nécessite une intervention ciblée au sein les populations atteintes autour des facteurs sociaux du virus de l'immunodéficience humaine (VIH) ou du SIDA afin d'éviter la détérioration sociale et culturelle ainsi que le désavantage financier dont sont victimes les familles accueillant les orphelins du SIDA et les enfants eux-mêmes. Ces résultats pourraient amener à prendre des mesures relatives à la prévention du VIH/SIDA et au développement de stratégies plus générales afin d'atténuer l'impact de la maladie dans les familles et communautés rurales de Tanzanie et des parties du monde similaires.

Translated from English version into French by Alexandra Das Neves, through

TRANSLATORS WITHOUT BORDERS

Стратегии, используемые приемными семьями для преодоления и смягчения воздействия усыновления детей, родители которых умерли от СПИДа, в регионе Мбея в Танзании

Нелсенсиус Клау Фаук, Силвано Эдсон Муакиньяли, Лилиан Муанри

Краткое изложение

Цели: Целью данного исследования является рассмотрение стратегий, используемых приемными семьями детей, родители которых умерли от СПИДа (синдрома приобретенного иммунодефицита), для преодоления и смягчения воздействия СПИДа, в сельском районе Мбея в Танзании.

Методы: При проведении исследования для сбора данных использовался метод качественного опроса в форме подробных бесед один на один. Респондентами были главы семей (мужчины и женщины), осуществляющих основную опеку над детьми, чьи родители умерли от СПИДа, в сельском районе Мбея в Танзании. Для обработки данных использовался логико-структурный подход.

Результаты: Приемные семьи, осуществляющие основную опеку над детьми, чьи родители умерли от СПИДа, сталкиваются с различными трудностями, в том числе финансовыми, возникающими в результате повышенных расходов на медицинское обслуживание и продукты питания. Другие формы воздействия СПИДА на приемные семьи также включают недостаточные возможности трудовой деятельности и ограниченное время на преодоление возникающих трудностей. Для смягчения воздействия этих трудностей приемные семьи используют ряд стратегий, в том числе продажу собственности семьи и сдачу в аренду земли, пригодной для обработки, для получения дополнительных средств. Перераспределение некоторых ролей на усыновленных детей также используется в качестве стратегии смягчения воздействия и включает снятие детей с регистрации в школе с целью их участия в приносящей доход деятельности и поддержке семьи и ее потребностей. Создание дополнительной деятельности, приносящей доход, например, птицеводческие проекты, было следующим механизмом адаптации и получило поддержку негосударственных и государственных организаций, в том числе Isangati Agricultural Development Organization (IADO) и органов местного самоуправления соответственно.

Заключение: Данные результаты предоставляют информацию о сложностях, с которыми сталкиваются приемные семьи детей, родители которых умерли от СПИДа, в сельском районе Мбея. Признание существования этих проблем требует направленных вмешательств, необходимых для устранения социальных детерминант, лежащих в основе ВИЧ и СПИДа среди затронутых групп населения, для предотвращения дальнейшего усугубления социальных, культурных и экономических неблагоприятных условий для семей, осуществляющих опеку над детьми, родители которых умерли от СПИДа, и для самих сирот. Эти результаты могут оказаться полезными при обсуждениях, ведущих к профилактике ВИЧ/СПИДа и разработке более широких стратегий смягчения негативного воздействия на семьи в сельской местности в Танзании и подобных условиях по всему миру.

Translated from English version into Russian by Elena McDonnell, through

TRANSLATORS WITHOUT BORDERS

Estrategias de las familias adoptivas para hacer frente y aliviar el impacto de los niños huérfanos por el SIDA en la región de Mbeya en Tanzanía

Nelsensius Klau Fauk, Silivano Edson Mwakinyali, Lillian Mwanri,

Resumen

Objetivos: la finalidad del estudio era conocer las estrategias empleadas por las familias adoptivas de niños huérfanos a causa del Síndrome de Inmunodeficiencia Adquirida (SIDA) para hacer frente y aliviar el impacto del SIDA en esos grupos familiares en el distrito rural de Mbeya, Tanzanía.

Métodos: el presente estudio utilizó un estudio cualitativo de recolección de datos a través de exhaustivas entrevistas individuales. Los entrevistados fueron hombres y mujeres jefes de familia que prestaban cuidados esenciales a niños huérfanos por el SIDA en el distrito rural de Mbeya en Tanzanía. Para el análisis de los datos se utilizó el enfoque marco.

Resultados: las familias adoptivas de huérfanos por el SIDA tuvieron que hacer frente a varios problemas que incluían dificultades económicas para afrontar el aumento de los gastos de asistencia médica, y la mayor demanda de alimentos dentro del núcleo familiar. Asimismo, tenían dificultad para encontrar empleo y escaso tiempo para resolver los problemas emergentes. Para mitigar esos desafíos, y a fin de obtener ingresos adicionales, las familias adoptivas de huérfanos por el SIDA se valieron de una variedad de estrategias para hacer frente a las circunstancias, entre ellas, la venta de bienes familiares y el alquiler de parcelas de tierra cultivable. Asimismo, reasignaron las tareas para que los niños huérfanos por el SIDA sirvieran como mano de obra para contribuir a aliviar las dificultades. Esto entrañó la participación de los niños no matriculados en la escuela en actividades generadoras de ingresos para responder a las necesidades familiares de ingresos adicionales. La creación de actividades de generación de ingresos adicionales, tales como los proyectos de cría de aves de corral, fueron otros mecanismos de ayuda que recibieron el apoyo de las organizaciones no gubernamentales y gubernamentales, por ejemplo, de la Organización de Desarrollo Agrícola Isangati (IADO) y del gobierno local, respectivamente.
Conclusiones: estos hallazgos proporcionan información sobre los desafíos a que deben hacer frente las familias adoptivas de huérfanos por el SIDA, así como los propios niños huérfanos a causa del SIDA en el distrito rural de Mbeya. El reconocimiento de estas cuestiones exige intervenciones específicas que son necesarias para abordar los determinantes sociales subyacentes del Virus de Inmunodeficiencia Humana o VIH y el SIDA en las poblaciones afectadas, con miras a prevenir un mayor deterioro del perjuicio social, cultural y económico impuesto a las familias que tienen a su cuidado niños huérfanos por el SIDA, así como a los propios niños afectados. Estas conclusiones podrían ser útiles para promover el debate y podrían llevar a la prevención del VIH/SIDA y la elaboración de estrategias de mitigación más amplias para aliviar el impacto en las familias rurales y las comunidades en Tanzanía y en entornos similares en todo el mundo.

Translated from English version into Spanish by M. Kaszczynice, through

TRANSLATORS
WITHOUT BORDERS
